# Membranous Obstruction of the Larynx

**Published:** 1900-02

**Authors:** W. Jay Bell

**Affiliations:** Atlanta, Ga.


### MEMBRANOUS OBSTRUCTION OF THE LARYNX.*

BY W. JAY BELL, M.D.,

ATLANTA, GA.

I feel that we have had discussion enough to-night and was in hopes that we would adjourn. I stand before you without a paper in hand. I will have something to say to-night in the line of membranous obstruction of the larynx. For convenience I will divide the subject into two forms: that of the catarrhal inflammatory form, not a true membrane but exudative, and that of a true pseudo-membrane, due to the Klebs-Lœffler bacillus—-diphtheritic.

The first form, that of simple catarrhal inflammatory exudation, is, as I hold, not a true pseudo-membrane, and is due, not to the diphtheria bacillus, but to the streptococcus as a rule, the streptococcus being found most prolific. This form gives us, however, as much serious obstruction as the form caused by the Klebs-Lœffler bacillus or diphtheritic. Therefore it has become the custom to refer to this as membranous croup.

The second form, that of true diphtheria, is the real pseudo-membrane and the only one that we have. This is the most frequent cause of laryngeal obstruction. The point of diagnosis between these forms is really in many cases a very difficult one,

*Remarks made before Atlanta Society of Medicine.

and of the two forms of obstruction, that of the inflammatory and that of diphtheritic, it is exceedingly difficult and often cannot be determined even by the microscope. However, the fact that we have in a given case the Klebs-Lœffler bacillus is conclusive; though we often have the obstruction clearly membranous, that is, exudative in character, or even diphtheritic in character, without being able to find the bacillus of Lœffler, there being no membrane in reach or any area from which the culture could be taken. Therefore the absence of the bacilli is not conclusive that we have not diphtheria. Their presence however makes the matter clear without doubt. We therefore make this examination and repeat it as often as is reasonable, until fully satisfied that we have not a diphtheritic form. We are too frequently confronted with cases of obstruction of the larynx that are not membranous but simply catarrhal. I refer to this simply because it so often makes our diagnosis very difficult. The differential diagnosis, however, may be gotten at in a little time, but in the first twenty-four hours of the attack it is often very difficult to make a positive diagnosis. The fact that you have no membrane in sight is not conclusive that you have not a diphtheritic or inflammatory exudation in the larynx. However, in the catarrhal form we will usually have relaxed periods in which the dyspnea is not so marked. Further measures will sometimes clear up the diagnosis. By the use of relaxants we will have a relaxation of the spasmodic form that we usually have in the catarrhal. It is therefore not always a matter of difficulty. But in some cases it is so severe that we are unable to decide, and they pass on to a very serious condition, at times requiring intubation.

The treatment of these forms of laryngeal obstruction is a matter that I will not dwell very largely upon other than the mechanical relief. The simple measures are so familiar that I deem it unnecessary to mention them. The point of greatest interest, it seems to me, is that of cases of so serious a nature as to require further measures than simple medical treatment. These cases often pass into a condition of dyspnea, and I feel that the physicians very often wait on these cases until they have exhausted largely the strength of their patients instead of giving them the relief that can be had by the use of O'Dywer's tube. In the diphtheritic form of course the antitoxin is to be used and at once, but this is not going to relieve the responsibility of the physician in not going ahead and relieving the dyspnea, which will exhaust the strength of the patient. We cannot wait on a remedy to absorb the membrane which is strangling the child. For relief we can use mechanical measures, and the question is what to use, whether tracheotomy or intubation? In all these cases it is clear to my mind that intubation is to be preferred. It can be done in five to eight seconds, and if the tube gives relief it can be worn with comfort, and in the majority of cases it is successful. At times it is necessary to do tracheotomy where the tube does not reach below the obstruction, but this occurs in only a few cases.

In cases of obstruction of the larynx we often wait, hoping intubation will not be necessary, and thereby allow our patient to lose largely in strength and cripple the heart in such a way that our result is then hazarded when the tube is demanded. Therefore, I claim that when the child is making an effort to obtain oxygen sufficient it is not necessary to wait for cyanosis. Intubation is the best thing that can be done, and it should be done early rather than wait, hoping the membrane may be absorbed or expelled.

### DISCUSSION.

*Dr. Hubbard:* I have nothing much to say on the subject, as I have not treated much diphtheria. I have had some cases of spasmodic croup and some of true diphtheria. I have used the antitoxin twice. In one case the trouble was laryngeal at the beginning.

I saw the case first at eight o'clock in the evening, a child ten months old, with a temperature of 105 and marked dyspnea at the time. I did not think it a fair test for the antitoxin but immediately decided to try it. I called Dr. Todd into consultation as I had never used it before. I gave it one hour after I saw the child. I saw it again that night at eleven o'clock and again the next morning, and it was in a state of exhaustion and with marked dyspnea. I would have liked to try the intubation in that case, but I doubt whether it would have been of any benefit, as the intoxication was so very profound.

The other case I was called to one evening and I diagnosed as diphtheria. There was a little patch of membrane on the tonsils and a temperature of 103.5. Dr. Cromer made examination with the microscope and he decided it was diphtheria. I gave the antitoxin and the temperature was reduced next day to 102. The temperature went on down and the child made a rapid recovery and was up in a week.

I believe with Dr. Bell that intubation is a good thing. I think it carries less prospect of serious result, and more mothers will consent to it than to tracheotomy. I think I shall adopt it in the future.

*Dr. Murphey:* There is one point in intubation that Dr. Bell failed to bring out. In some cases there is danger of driving the membrane downward and blocking up the trachea, and it will then have to be removed. In other cases where the membrane is peeling the tube will also block up the passage. This is always to be taken into consideration. However, I believe in intubation and have used it once and I think the life of the child was saved by its use.

*Dr. Visanska:* I think intubation is a good thing and far ahead of tracheotomy, whether the obstruction is diphtheritic or otherwise. I saw Dr. Bell relieve a case once. The dyspnea was relieved, but the child had absorbed so much poison that it lived only a few hours. It also had a double pneumonia. I believe the tube had to be removed once and was then reinserted. In tracheotomy there is danger of infecting the wound which you make. Intubation is a great relief to the child and to the parents.

*Dr. Bell* (closing the discussion): As to my experience with intubation since coming to Atlanta, I will not go into details, but I have done fourteen intubations, and of these there were four fatalities. One of these was due to a deep extension of the membrane and another was apparently capillary bronchitis in which the tube was clear and open. The other two cases which were fatal were complicated with pneumonia.

One point which I intended to mention in regard to treatment before getting to the point of doing the intubation, was the use of calomel fumigations every two or three hours. The relief of dyspnea from this is remarkable. I simply put a tent over the child and give the inhalations for fifteen minutes out of every two hours, and in this way you get a coating on the larynx that is of great relief. This cannot be used after the tube is in place. Instead of this you should use a steam spray of menthol and turpentine and keep the air of the room moist with it.

As to the point mentioned about the tube pushing the membrane down, I have seen this in a few cases, but the immediate removal of the tube causes the expulsion of the loosened portion of the membrane. It acts simply as the old operation of forcing something down into the larynx to break loose the membrane. Therefore this is not an objection, as it removes the membrane and the tube may not then have to be worn. I saw one case which was fatal from a loose portion of the membrane at the lower end of the tube, causing a valvular closure, but this was due simply to a bunglesome measure on the part of the nurse by going for a former physician of the hospital staff who did not know how to use the tube, I being at dinner at the time. He tried to get the tube out but failed. In order to avoid any difficulty in removing the tube I always leave a silk thread in the tube and any one can then remove it, as otherwise the child might die of strangulation while waiting for me to reach the room. I instruct the attendant to lift the tube by passing the finger under the cord, then with cord tense raise finger to roof of the mouth and disengage the tube. I instruct them that this is to be done only when it is found that the child cannot breathe. I find that when the tube has been worn for a few hours the obstruction does not return for at least a few hours.

